# A New Intraoral Six-Degrees-of-Freedom Jaw Movement Tracking Method Using Magnetic Fingerprints

**DOI:** 10.3390/s22228923

**Published:** 2022-11-18

**Authors:** Kinta Morikawa, Ryosuke Isogai, Junya Nonaka, Yoshifumi Yoshida, Shugo Haga, Koutaro Maki

**Affiliations:** 1 Department of Orthodontics, School of Dentistry, Showa University, 2-1-1 Kitasenzoku, Ota-ku, Tokyo 145-8515, Japan; 2 Research and Development Department, Seiko Holdings Corporation, 563, Takatsukashinden, Chiba 270-2222, Japan

**Keywords:** six degrees of freedom, jaw movement, magnetic sensor, orientation sensor, position estimation, three-dimensional reconstruction

## Abstract

We proposed a novel jaw movement tracking method that can measure in six degrees of freedom. The magnetic field generated by a permanent magnet paired with a small, low-power-consumption Hall effect magnetic sensor is used to estimate the relative distance between two objects—in this instance, the lower and upper jaws. By installing a microelectromechanical system (MEMS) orientation sensor in the device, we developed a mouthpiece-type sensing device that can measure voluntary mandibular movements in three-dimensional orientation and position. An evaluation of individuals wearing this device demonstrated its ability to measure mandibular movement with an accuracy of approximately 3 mm. Using the movement recording feature with six degrees of freedom also enabled the evaluation of an individual’s jaw movements over time in three dimensions. In this method, all sensors are built onto the mouthpiece and the sensing is completed in the oral cavity. It does not require the fixation of a large-scale device to the head or of a jig to the teeth, unlike existing mandibular movement tracking devices. These novel features are expected to increase the accessibility of routine measurements of natural jaw movement, unrestricted by an individual’s physiological movement and posture.

## 1. Introduction

Human mandibular movement is expressed as a series of complex movements involving the interaction of three-dimensional, rotational, and translational movements. Individual variations in mandibular movement arise because of the morphology and alignment of the temporomandibular joint, ligaments, muscle groups, and teeth, some of which can prevent normal jaw movement [1,2,3]. Thus, evaluating mandibular movement is essential in the functional assessment of the temporomandibular joint with regard to changes in facial morphology and occlusion associated with orthodontic treatments. Furthermore, in general dental treatment, understanding mandibular movement to detect temporomandibular disorders is equally important. The prevalence of temporomandibular disorders is very high, and the manifestation of these disorders can range from being asymptomatic to expressing debilitating symptoms. Epidemiological studies indicate that 50–60% of the general population has some sign of masticatory system dysfunction [4].

Information necessary for diagnosing temporomandibular disorders includes the trajectory of mandibular movement and the amount of mouth opening. Thus, instruments for measuring mandibular movement are essential for recording and diagnosing mandibular movement, characterized by restricted or abnormal trajectories of the temporomandibular joints. Existing instruments for mandibular movement tracking include the K7 device (Myotronics-Noromed, Inc., Kent, WA, USA), which senses and tracks a magnetic field generated by a magnet [5]; the Gnatho-Hexagraph II (GC Corporation, Tokyo, Japan), which uses a camera to trace light emitted by light-emitting diodes [6]; and the WinJMA System (Zebris Medical, GmbH, Reutlingen, Germany), which uses ultrasonic distance measurement [7]. These tracking methods can obtain a stable reference point for ranges, thus providing stable and precise data because the sensor is placed extraorally. However, various tracking devices each have different disadvantages that can benefit from improvement. For example, the magnet in the K7 is fixed to the labial aspect of the mandibular anterior teeth and requires the placement of a large measurement device—called the sensor array—on the head. The Gnatho-Hexagraph II device and WinJMA System require the fixation of a large jig to the outer surface of the mandibular anterior teeth to extract the jaw movement out of the mouth mechanically, and sense ultrasonic wave or guide light at a distance from patients. Therefore, the weight of the jig and sensor generates secondary moment at the time of opening and closing the mouth, unintended moment in the direction of mandibular opening, and shaking. Furthermore, a common drawback of these tracking instruments occurs in patients with an overbite or excessive salivation, in which case, using dental resin or adhesive tape to fixate jigs or magnets onto the labial aspect of the mandibular anterior teeth can be difficult. Moreover, wearing an extraoral device restricts a patient’s physiological movement and posture, which would prevent the device from accurately tracking the patient’s natural mandibular movement in detail.

In addition to the commercial products listed previously, other mandibular movement tracking devices, operating on various principles, have been reported. According to a recent review, several methods (such as extraoral extraction and measurement of jaw movement using mechanical linkage, mandibular position estimation using magnetism, and motion detection using video or radiographs) still require large extraoral devices [8], indicating that completing measurements in the oral cavity is difficult and they cannot be used as a portable and simplified jaw tracking device. This is a significant issue in order to measure jaw movement in daily living activities and contributes to self-care relative to the jaw functions such as masticatory system dysfunction. Among the various aforementioned methods, magnetic measurements are promising for intraoral measurable jaw trackers in terms of low power consumption and miniaturization. In 2002, Flavel et al. [9] fixed a metal bracket with acceleration and magnetic sensors onto the labial surface of the maxillary and mandibular incisors to evaluate the vertical position of the lower jaw in relation to the upper jaw. However, they only measured the position of the lower jaw to one degree of freedom (i.e., up and down), and only used the mean data of eight measurements to reduce noise so that the device could not measure the trajectory of individual jaw movements. Furthermore, it required a 75V 50 mA power supply, thereby preventing adaptation to a portable version. Fuentes et al. [10] examined Posselt’s diagram and Gothic arch by measuring individuals’ jaw movement using an electromagnetic articulograph that requires large instruments. Moreover, Nishigawa et al. [11] indicated that the feature of mandibular movements could be determined by electromyography. However, electromyography requires extraoral electric potential sensors. Jucevičius et al. [12] measured mandibular movement using a magnetic sensor attached to the molars on a robot, but the team did not clinically test it in humans. Furthermore, their model does not obtain sufficient information to evaluate human jaw movement because it lacks a component for measuring jaw rotation.

In this study, we proposed a mandibular movement tracking method that can measure in six degrees of freedom without fixing the head or being compromised by the patient’s occlusal state or saliva and it is effective as a mouthpiece-type portable device. In the proposed method, the movement of the lower jaw can be measured with a simple compact system comprising a magnetic sensor, an inertial measurement unit, and a magnetic sensor without fixing a jig on the dental surface, generating moment, or restricting the patient’s physiological movement and posture.

## 2. Materials and Methods

### 2.1. Measurement Principles

**Figure 1:**
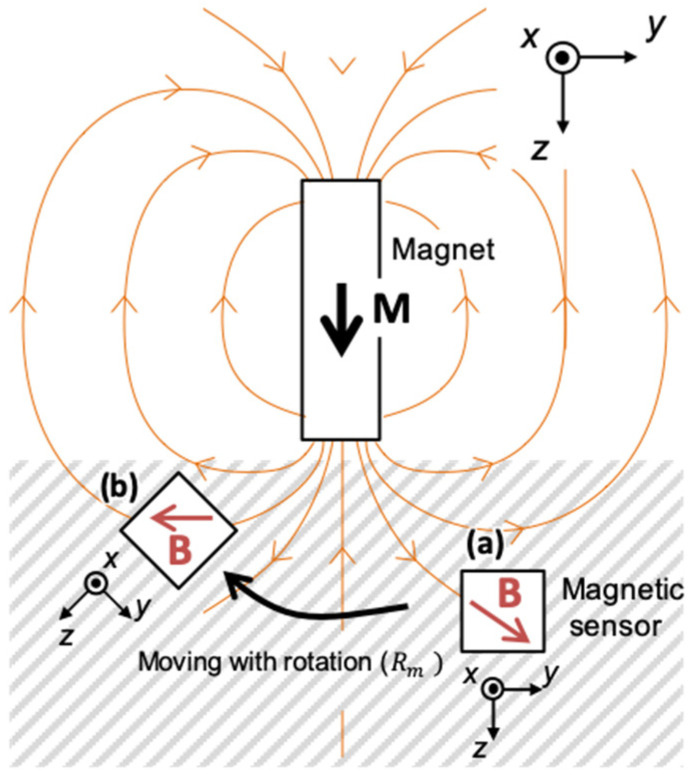
Principle of positional estimation.

Figure 1 presents the principles of magnetic position estimation in this paper. To track lower jaw movement, the magnetic flux density vectors along the path of displacement in the spatially distributed magnetic force is measured to estimate the trajectory of the movement. A similar method of magnetic positional estimation uses an alternating magnetic field. This method has benefits such as resistance to disturbance and the ability to capture fine changes to magnetic flux density; however, it requires a coil for excitation and a constant excitation current, which hinders miniaturization or ensuring a long battery life [13]. Therefore, the present device uses a permanent magnet that generates a magnetic field that can be miniaturized for tracking intraorally. Using this method allows the generation of a relatively large magnetic field for its small volume without requiring external energy supply for excitation. As shown in Figure 1, the magnetic flux density vectors of the static magnetic field generated by a magnet varies with the direction and distance from the magnet. For example, when the lower jaw moves from point (a) to point (b), by using the unique correspondence between the position and the magnetic flux density at that location, a trajectory can be obtained by continuously measuring the magnetic flux density. Therefore, measuring the three-dimensional relative position with respect to the magnet is possible, based on this principle, by attaching a magnetic sensor that can measure the three-dimensional magnetic flux density vectors to the lower jaw.

The static magnetic field generated by the magnet is measured by the magnetic sensor at the initial position (a), and the displacement is estimated by measuring the three-dimensional magnetic field as a result of the parallel shift with the rotation operation expressed by the rotation matrix *R*_m_ (b).

**Figure 2:**
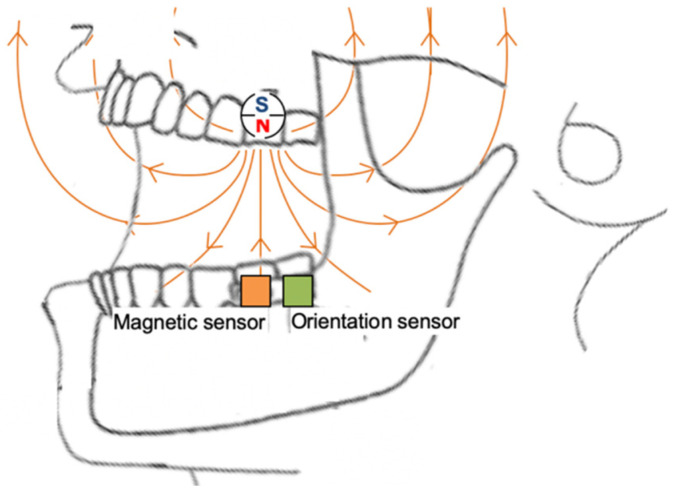
Layout of the components in the proposed system.

Figure 2 shows the components of the system that tracks the positions of the upper and lower jaws, based on this method. Generally, jaw movement is expressed as movement of the lower jaw with respect to the upper jaw. Thus, the magnet that provides the reference is placed on the upper jaw and the magnetic sensor is placed on the lower jaw to measure the changes to magnetic flux density associated with the positional changes in relation to each other. Similar existing methods used to estimate position by magnetic flux density are based on the theoretical calculations of a magnetostatic field generated by a magnet. However, the effects of various factors, such as magnet shape, material properties, and surface treatment and roughness on the measured outcomes are difficult to control. Precise estimates of position to within several millimeters, which was the aim in this study, are accordingly unachievable when using existing methods [14].

The magnet is placed in the upper molar, and the magnetic and orientation sensors are placed in the lower molar.

Thus, the current study used a method—hereinafter referred to as the “fingerprint method”—that collects the distribution of the magnet’s three-dimensional magnetic flux density vectors experimentally in advance [15,16]. The fingerprint method results in an accurate magnetic flux density distribution of the magnetostatic field generated by an actual magnet, which is difficult to obtain by theoretical calculations alone, as explained previously. Specifically, the fingerprint method uses data (i.e., fingerprints) obtained by acquiring the correspondence between the position and the magnetic flux density vectors while changing the XYZ position of the magnet and magnetic sensor. This allows the map f:P→B, which shows the correspondence between set B of magnetic flux density vectors corresponding to set P=X×Y×Z of relative positions with respect to the magnet, obtained by the actual measurement. X*,* Y*,* and Z is the set of the individual XYZ components of the relative position when obtaining the fingerprint. The map, *T*, is configured by converting the magnetic flux density vectors to relative positions, using the fingerprints:

(1) $$T_{x}:B\to X,\;T_{y}:B\to Y,\;T_{z}:B\to Z$$

Maps *T_x_, T_y_,* and *T_z_* are functions that return *x*, *y*, and *z,* corresponding to the magnetic flux density, $B\left(x,\;y,\;z\right),$ of the fingerprint mentioned previously. When assigning magnetic flux density, points other than those where fingerprints have been obtained, the relative positions that have been interpolated are returned. Interpolation is achieved by triangulating the magnetic flux data of the fingerprint by using Qhull and performing a linear barycentric interpolation on each triangle [17].

The healthy lower jaw moves with parallel displacement and tilts with respect to the upper jaw. Thus, the magnetic flux density vector measured by the magnetic sensor is likewise measured with tilt. At this time, the fingerprint obtained previously is measured at the fixed angle and the tilt is not taken into account. Therefore, a simple estimation of position using the aforementioned interpolation will result in major diversions from the preconditions and consequently major errors. Therefore, the following rotation is performed to correct the tilt of the magnetic flux density vector used for position estimation:

(2) $$B^{\prime }\left(t\right)=R_{m}^{-1}\left(t\right)B\left(t\right)$$

*R_m_*(*t*) is the rotation matrix representing the orientation of the magnetic sensor at time *t* with respect to the orientation at the time of obtaining the fingerprint. The density vector is observed as a rotation of equal magnitude as the tilt of the magnetic sensor. Therefore, inverting the rotation allows the vector to be obtained without rotation. *B*′ thus obtained can be used to estimate position so that the parallel displacement of the lower jaw can be accurately detected, despite mandibular tilt. The rotation matrix *R_m_*(*t*) is obtained using the orientation sensor placed close to the magnetic sensor (Figure 2). Using an orientation sensor enables accurate measurement of the relative position with mandibular tilt and obtains three-dimensional information on the mandibular orientation so that mandibular movement can be measured at six degrees of freedom (i.e., three dimensions of parallel displacement and three dimensions of rotation).

**Figure 3:**
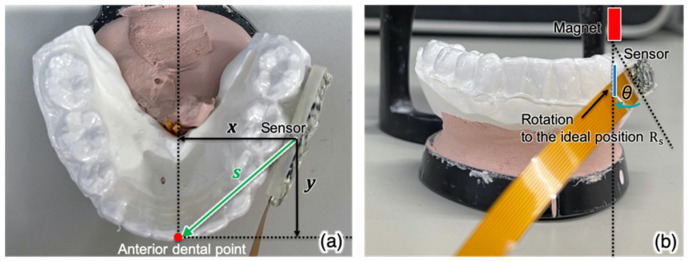
Translation of data from the molar to the anterior dental point. (**a**) Superior view; (**b**) frontal view.

The trajectory of the mandibular anterior teeth is generally used in dental clinical practice. This device can be placed on other teeth and still generate the trajectory of the front teeth by translating values, as long as the geometric details of the oral anatomy are known. As shown in Figure 3, parallel displacement at the anterior dental point due to the tilt of the lower jaw is added to the position after correction of the tilt of the magnetic sensor. That is,

(3) $$x^{\prime }\left(t\right)=R_{s}x\left(t\right)+\left\{R_{m}\left(t\right)-1\right\}s$$

in which *R_s_* is the rotation matrix representing the tilt of the magnetic sensor with reference to the initial positional relationship at the time of fingerprint acquisition, and *s* is the vector from the point of magnetic sensor installation to the front tooth point. Individual differences from these constants exist because of variations in dentition and shifts at the time of sensor installation; therefore, they should be measured in advance via devices such as intraoral 3D scanners.

**Figure 4:**
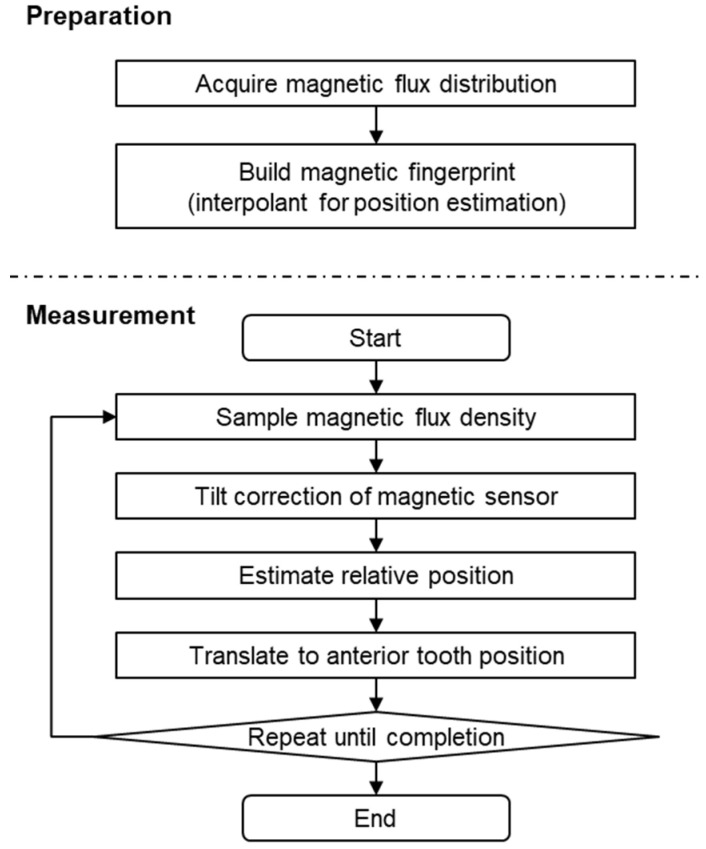
Measurement and data processing flow diagram.

Figure 4 presents the main measurement and data processing flow. Before measuring jaw movement, the present methods must collect the magnetic fingerprint. As mentioned earlier, the magnetic flux density vector distribution generated by the magnet is collected to generate a complement function for position measurement. In the jaw tracking step, the magnetic flux density vector at a given point in time is observed with a magnetic sensor placed on the lower jaw, and the position is estimated using the fingerprints collected in advance after tilt correction. When the sensor is placed on any tooth other than the front teeth, the data collected from that location are converted geometrically to correspond to the movement at the front teeth. This action is repeated until the measurement for the jaw movement is completed (i.e., until the desired number of opening and closing movements has occurred).

The fingerprint method can be used to translate the data of relative position sensing, obtained from a compact and comfortable device placed on the molars, into data of the jaw movement trajectory from the perspective of the front teeth, which has clinical significance. The following sections will present the quantitative assessments of the efficacy of this method and demonstrate how jaw movement can be measured with this novel device.

### 2.2. Assembly of the Sensing Module

#### 2.2.1. Sensing Module and Measurement System

**Figure 5:**
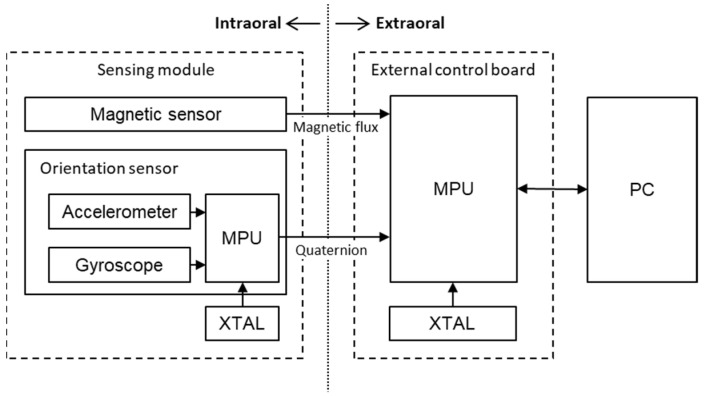
Block diagram of the tracking system. MPU, memory protection unit; XTAL, crystal; PC, personal computer.

Figure 5 presents the block diagram of the measurement system. The tracking system comprised the sensing module, external control board, and computer (Windows 10; Microsoft, Redmond, WA, USA). The sensing module consists of a magnetic sensor, which measures magnetic flux density (AK09916; Asahi Kasei Microdevices Corp., Tokyo, Japan), and an orientation sensor (BNO055; Bosch Sensortec GmbH, Munich, Germany).

Various types of magnetic sensors exist that are ideal for mandibular motion estimation, and the appropriate one must be selected. Several studies that have estimated the position of a living body using Hall effect magnetic sensors [12] or magnetoimpedance (MI) sensors [18,19]. The most important requirement in our configuration is that the sensor can effectively detect ±3 mT, the dynamic range of the magnetic field generated by the small permanent magnet during mouth opening and closing. One candidate is the fluxgate sensor, often used in precision medical equipment, but although it has excellent sensitivity, it is large and its detectable magnetic field range is 0.02 mT or less, making it unsuitable for measuring magnetic fields of ±3 mT; its high sensitivity cannot be used in this configuration. Conversely, Hall sensors, magnetoresistive (MR) sensors, and MI sensors, have a detectable magnetic range of above 3 mT. Among them, Hall sensors are inferior in sensitivity and resolution to other magnetic sensors, but they have a small package, low power consumption, and are readily available at low cost because they are the most commercialized [20]. In this research, since a relatively large magnetic field of about several mT was detected near a magnet, the sensitivity and resolution suitable for this are important, therefore a compact and power-saving Hall sensor was comprehensively evaluated to achieve intraoral operation.

In our sensor module, the magnetic Hall sensor measures three-dimensional magnetic flux density with a sensitivity of 0.15 µT in the range of ±4900 µT and is built into an integrated sensor module (ICM-20948; InvenSense, San Jose, CA, USA) with a size of 3 × 3 × 1 mm. The orientation sensor consists of an acceleration sensor and gyroscope and can measure acceleration of 0.01 G in the range of ±2G and angular velocity of 0.2°/s in the range of ±125°/s. It calculates and outputs a quaternion from acceleration and the tracking results of each angular acceleration. The orientation sensor’s dimensions are 3.8 × 5.2 × 11.3 mm.

**Figure 6:**
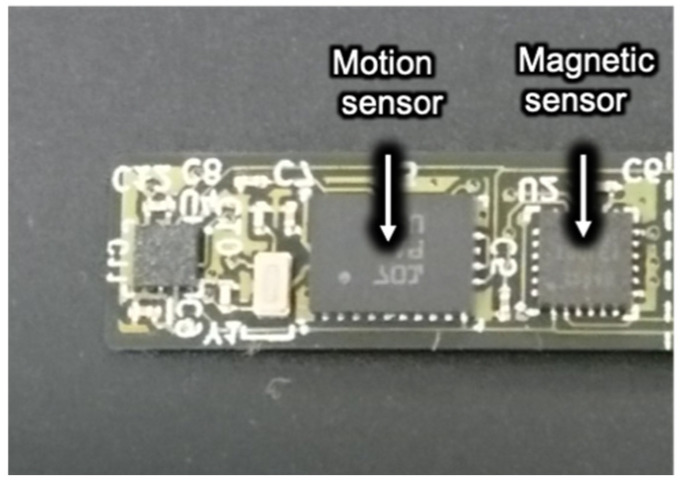
Photograph of the sensor module, including a magnetic and a motion sensor.

Figure 6 displays the sensing module with sensors. The compact design of the module (7.0 mm H, 23.0 mm W, and 2.5 mm D) allows it to fit along an individual’s dentition. The sensing module is connected to the external control board by a cable and controlled by the microcontroller by a twin wire interface.

A permanent magnet is used to generate the magnetostatic field measured by the magnetic sensor. The magnet fixed to the mouthpiece must be thin to reduce patient discomfort. The samarium–cobalt magnet, selected because of its high surface magnetic flux density per unit volume, is 6 mm in diameter and 3 mm in thickness. Samarium–cobalt magnets are radially polarized and have a surface magnetic flux density of 300 mT. The surface of the magnet is coated with nickel, and the safety of nickel exposed to the oral cavity has been confirmed [21]. The mouthpiece-shaped device described in the next section is used to place the sensing module on the lower jaw and the magnet on the upper jaw.

#### 2.2.2. Manufacturing Method of the Mouthpiece-Type Mandibular Movement Measuring Device

**Figure 7:**
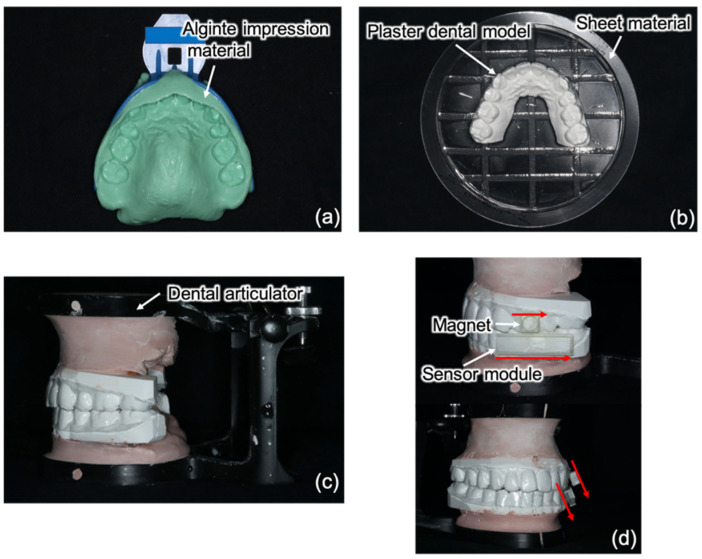
Manufacturing procedures of the mouthpiece-type mandibular movement-measuring device. (**a**) Impressions of the teeth are obtained; (**b**) the mouthpiece sheet material is attached; (**c**) the dental models are attached to the dental articulator; (**d**) the magnet and sensor module are installed parallel to each other.

The mouthpiece, mounted with the magnet, magnetic sensor, and orientation sensor, was custom-made for each patient. Based on the procedures described in a previous method [22], the mouthpiece is first formed by molding an alginate impression material to the upper and lower teeth (Aroma Fine Plus; GC, Corporation) (Figure 7a). Plaster (Zo-Stone; Shimomura Gypsum, Saitama, Japan) is then poured into a dental mold to create the dental model. After the plaster dental model hardened completely and was retouched, a 0.75 mm mouthpiece sheet material (Erkodur; Erkodent Erich Kopp GmbH, Pfalzgrafenweiler, Germany) was attached to the upper and lower plaster dental models and placed in a vacuum thermoforming unit (Erkopress ci motion; Erkodent Erich Kopp GmbH) (Figure 7b). After thermoforming, the upper and lower plaster dental models were further retouched and attached to the dental articulator (Figure 7c). An acrylic resin supportive material was attached on the buccal aspect of the first molar of the upper plaster dental model to fix the magnet. The process of fixing the sensor module with the acrylic resin was similarly repeated on the buccal aspect of the lower first molar. At this point, the magnet and sensors must be installed parallel to each other (Figure 7d).

Saliva that interferes with this orally worn mouthpiece can cause motion to be mistracked. Therefore, an electrical insulating material (approximately 20 μm thick) was used to coat the sensing module and a 0.3-mm-thick dental silicone (EXACLEAR; GC Corporation) was used to carefully cover the magnet and sensor to make them water resistant. The mouthpiece was then removed from the plaster dental model and completed by retouching and polishing its margins. The sensor used in this study was very light, weighing only 1.59 g, which is significantly lower than the weight of the sensor groups attached to the dental surface in the existing mandibular movement tracking devices K7 and the WinJMA System, (5.01 g and 66.37 g, respectively). The proposed sensor will dramatically reduce the effects of the sensor weight on jaw movement.

### 2.3. Mandibular Movement Tracking

#### 2.3.1. Precision Assessment of the Sensor Position

**Figure 8:**
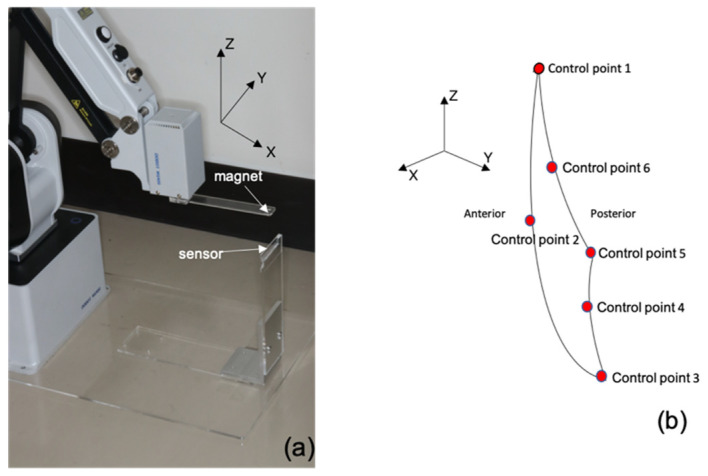
(**a**) Evaluation of the proposed method with the robot arm. (**b**) Posselt’s envelope composed of six control points as a model of a human jaw movement.

The magnetic fingerprints described in the Methods section were obtained by using the same procedure throughout the experiment. Acrylic plate jigs were attached to the end effector and base of the robot arm (DT-MG400-4R075-01; Yuejiang Technology Co., Ltd., Tokyo, Japan) to fix the samarium–cobalt magnet and magnetic sensor (Figure 8a). The X and Y directions were set to −25 to +25 mm, and the Z direction to 0 to +50 mm. Moving the samarium–cobalt magnet three-dimensionally in 1 mm increments generates the magnetic flux density vector in a mesh form. Approximately 90% of the points obtained was used to generate the interpolation function, which would be the fingerprint.

Furthermore, the remaining approximately 10% of points not used for the fingerprint were used to evaluate error of the position estimation of the area for which the fingerprint was obtained. Thus, we evaluated whether the position can be accurately estimated by using points other than those used for generating the interpolation function (i.e., unknown magnetic flux density data for the interpolation function). The root mean square error (RMSE) was used as an evaluation index.

In addition, the reference jaw motion generated by the aforementioned robot arm is used to quantitatively evaluate the precision of the sensor. We evaluated the accuracy of the robot arm in advance with a coordinate measurement machine (LEGEX9106; Mitutoyo Corporation, Kanagawa, Japan) and confirmed that the reproducibility of the standard jaw movement was within ±0.003 mm. The robot arm was controlled using six control points with reference to Posselt’s envelope of motion, which depicts the range of motion of the human mandibular anterior teeth during opening and closing movements, to define the standard jaw movement (Figure 8b) [23]. To determine the accuracy of positional precision of the sensing module, the standard jaw movement and mandibular movement calculated by the proposed fingerprint method were compared. The standard jaw movement was performed 10 times and the magnetic flux density was measured to estimate the position.

#### 2.3.2. Evaluation of the Mouthpiece-Type Jaw Movement Tracking Device

We compared the trajectories measured simultaneously by the mouthpiece-type mandibular movement tracking device and an existing approved mandibular movement tracking device (Digital JAW System; Zebris Medical GmbH) as a reference. The Digital JAW System is equipped with multiple ultrasonic sensors connected via a T-type attachment to the jaw. It tracks lower jaw position and tilt by measuring the propagation delays of externally delivered ultrasound by the multiple sensors. Based on the manufacturer’s published materials, the error of the WinJMA system is approximately 1.0 mm.

**Figure 9:**
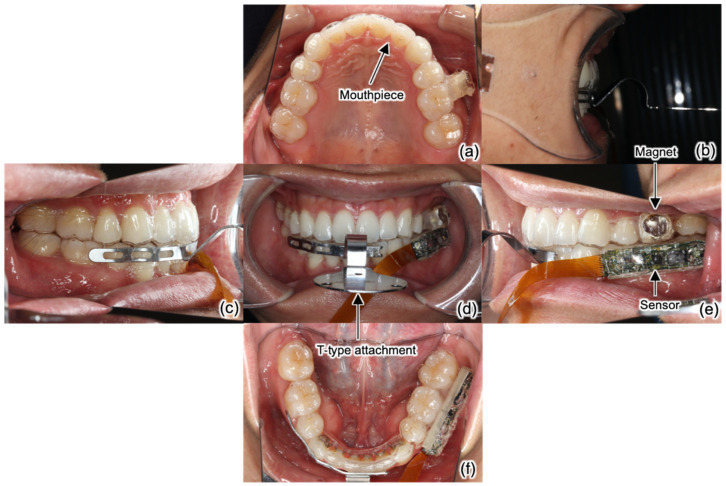
Intraoral photographs during simultaneous measurement with the present sensor and the WinJMA system. (**a**) Upper teeth; (**b**) overjet; (**c**) right lateral teeth; (**d**) frontal view; (**e**) left lateral teeth; (**f**) lower teeth.

**Figure 10:**
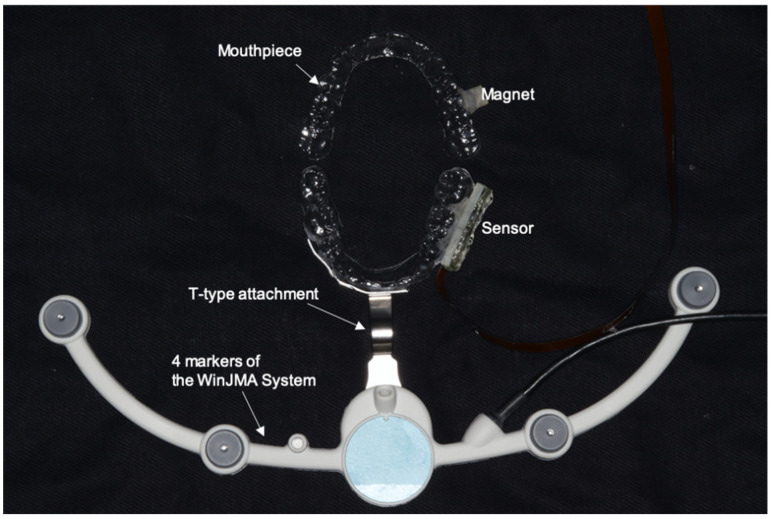
Setup of simultaneous measurement with the present sensor and the WinJMA system.

The T-type attachment of the Digital JAW System was attached to the front teeth portion of the lower jaw mouthpiece, as shown in Figure 9 and Figure 10. At the same time, the upper and lower mouthpieces with the sensor module are worn in the mouth. The four markers are attached to the T-type attachment and the microphone is fixed to the head. Simultaneous measurement with the mouthpiece-type mandibular movement tracking device and the Digital JAW System was started, the maximum mouth opening movements were performed three times. On account the data must be converted to the trajectory of the front teeth, XY (Figure 3a) and θ (Figure 3b), were measured from the individuals’ dental model. To convert the measured jaw movement to a three-dimensional movie, impressions of the individuals’ upper and lower dentition were taken with a digital impression scanner (TRIOS 3; 3Shape A/S, Copenhagen, Denmark) and converted to standard template library (STL) data. The three-dimensional tilt data obtained from the orientation sensor and three-dimensional parallel displacement data obtained from the magnetic sensor were then used to create a 3D image. 

## 3. Results and Discussion

### 3.1. Evaluation of the Sensor’s Positional Precision

**Figure 11:**
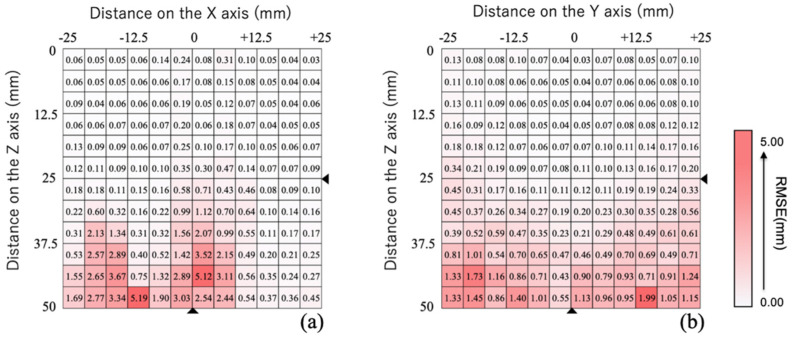
Errors of the estimated position for the (**a**) XZ and (**b**) YZ planes.

The results of the evaluation of positional precision in this proposed method are shown in Figure 11. The graphs show the distance between the magnet and magnetic sensor at 1 mm intervals on the horizontal X and Y axes set to −25 to +25 mm, and the vertical *Z*-axis to 0 to +50 mm. X = Y = Z = 0 is the point at which the magnet and magnetic sensor are closest to each other. The numbers in the cells show the RMSE at each position. On each axis, errors are smallest when the distance between the magnet and magnetic sensor is smallest, which may allow for precise positional estimation. This result is because the magnetic flux density measured increases as the magnet and magnetic sensor come closer together, thereby increasing the signal-to-noise ratio. The error was smallest at the following points: approximately 0.03 mm on the XZ plane (Figure 11a) near X = 25 and Y = 0, and approximately 0.03 mm on the YZ plane (Figure 11b) at X = 0 and Y = 0. Precision was lowest at approximately 5.0 mm on the XZ plane (Figure 11a), and approximately 2.0 mm on the YZ plane (Figure 11b). Distances over 40 mm between the magnet and sensor prevented a strong signal, thereby resulting in major errors. However, a precision of 0.03 mm to 0.47 mm or less can be expected when the distance between the magnet and magnetic sensor is within 25 mm at the time of jaw movement tracking, and 0.5 mm to 5.2 mm or less when the distance is ≤50 mm. Thus, sufficient accuracy of jaw movement tracking can be expected when the distance of the sensor is within 25 mm from the magnet.

**Figure 12:**
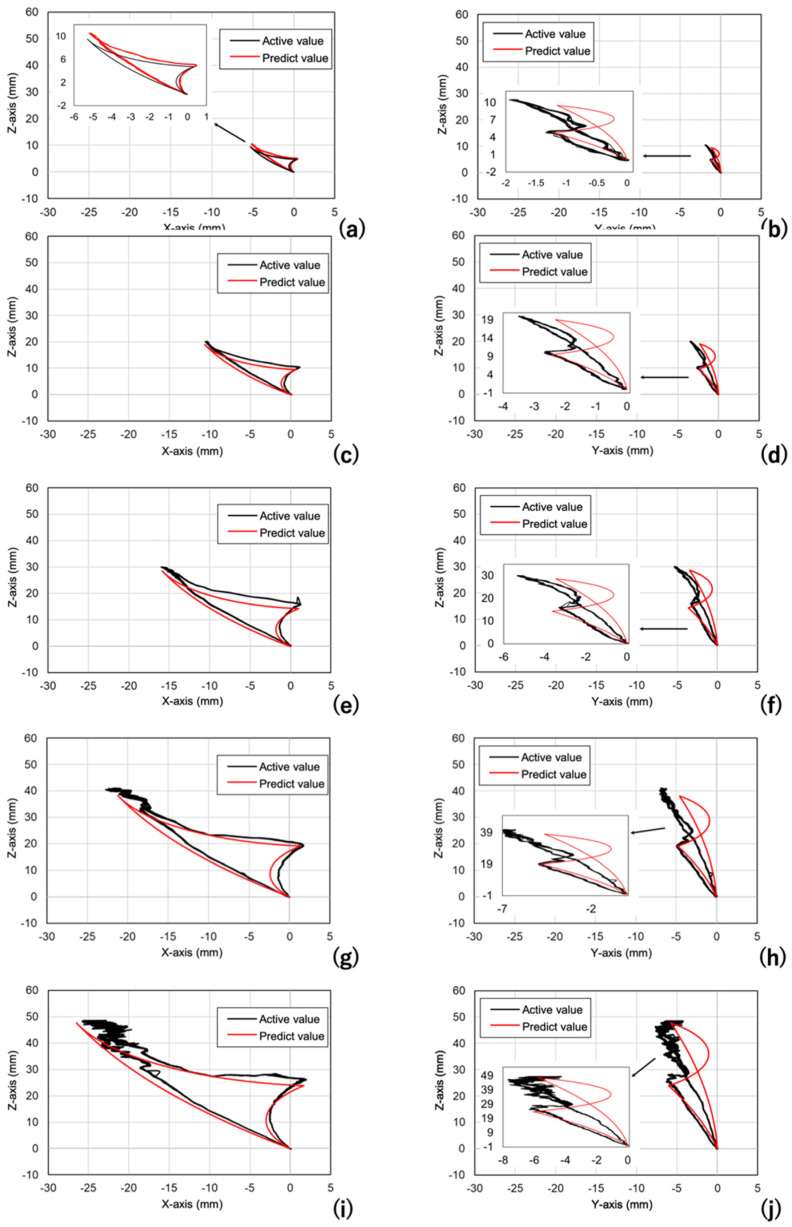
Comparison between the present sensor and the reference instruments. (**a**) Sagittal, 10 mm; (**b**) frontal, 10 mm; (**c**) sagittal, 20 mm; (**d**) frontal, 20 mm; (**e**) sagittal, 30 mm; (**f**) frontal, 30 mm; (**g**) sagittal, 40 mm; (**h**) frontal, 40 mm; (**i**) sagittal, 50 mm; (**j**) frontal, 50 mm.

**Figure 13:**
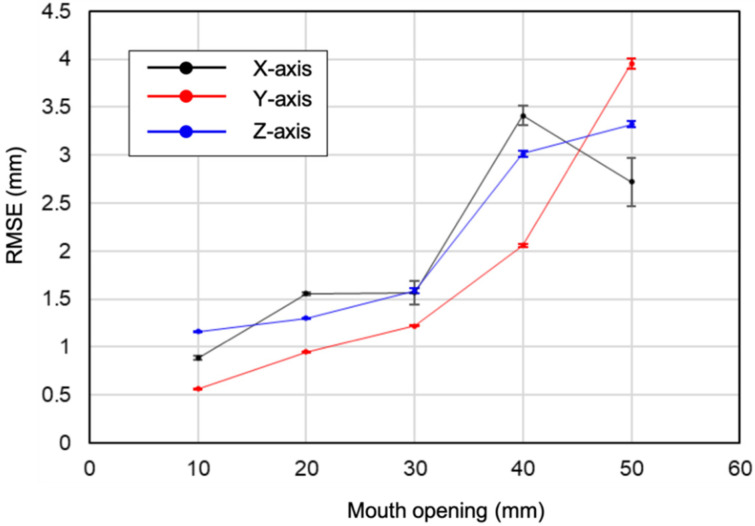
Root mean squared error (RMSE) calculated from the data of 10 trajectories of mouth opening.

**Figure 14:**
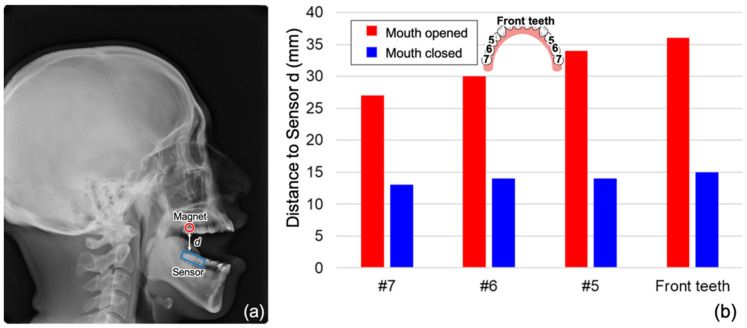
Distance between the magnet and magnetic sensor in each tooth, measured via cephalometric images. (**a**) The cephalogram. (**b**) The distance between the magnet and magnetic sensor varies with different tooth positions.

As a more practical method of evaluation, a quantitative assessment of precision was then conducted using movement replicating actual human jaw movement. The standard jaw movement was used with the maximum opening from 10 mm to 50 mm at 10 mm increments to evaluate the positional estimations in the XZ and YZ planes (the typical trajectories are shown in Figure 12). The trajectory in red is the reference data, and the trajectory in black is that of the present sensor. The RMSE calculated from the data of 10 trajectories allowed tracking within 1.5 mm error up to approximately 30 mm maximum mouth opening on the sagittal view (Figure 13). The maximum RMSE for mouth opening 40 and 50 mm was 3.3 and 4.0 mm, respectively. Distances between the magnet and magnetic sensor that exceeded approximately 30 mm on the sagittal view compromised the signal-to-noise ratio, which prevented the trajectory of the jaw movement from being depicted smoothly. The error was likewise between approximately 1.0, and 2.0 mm bilaterally when the distance from the sensor was approximately 30 mm or less in the frontal view. Above 30 mm, the errors of positional estimation increased, and the errors increased to ≥2.5 mm bilaterally. One solution to improve accuracy when opening the jaw more than 30 mm is to place magnets and sensors on the molars. Attaching the device at the molars generated the best results by minimizing the distance between the magnet and sensor near the position of maximum mouth opening. This result has been demonstrated in a previous study by Jucevičius et al. [12]. As a reference, the cephalogram (i.e., radiograph of the craniofacial area) of one volunteer was used to measure the distance between the magnet and magnetic sensor when the device was attached at the anterior, premolar (tooth #5), and molar regions (teeth #6 and #7), as shown in Figure 14. The distance was approximately 11 mm shorter when the device was placed at #7 than when it was placed on the anterior teeth. This translates to an estimated +11 mm of mouth opening that can be measured by placing the device on the molars rather than on the incisors.

### 3.2. Evaluation of the Mouthpiece-Type Mandibular Movement Tracker

Two male individuals with no jaw abnormalities, aged 30 years and 28 years, respectively, volunteered for this study. They had normal occlusion and no history of stomatognathic disorder or extreme trauma. This study was approved by the Institutional Review Board of the Showa University Dental Hospital (Tokyo, Japan; approval no. SUDH0080) and was conducted with the informed consent of the individuals.

**Figure 15:**
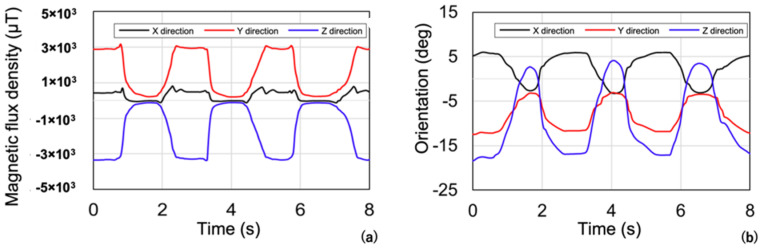
Time development of (**a**) magnetic flux density and (**b**) orientation of the mandibular jaw during the patient’s maximum mouth opening.

The magnetic flux density and orientation of this sensor from taking measurements are shown in Figure 15. The image shows three repetitions of maximum mouth opening. Magnetic flux density changes with the tilt and relative distance of the magnet and sensor; therefore, magnetic flux density is maximum when the mouth is closed. By contrast, the magnetic flux density approaches 0 on all three axes XYZ near maximum mouth opening. The geomagnetic field was 33 uT, which is regarded as noise that causes errors in position estimation, and the signal-to-noise ratio (SNR) is approximately 135 at mouth closing, while SNR is only 9 at the mouth opening, which is reflected as greater errors and scattering for mouth opening positions. With regard to the orientation, the X-axis, which is the axis that tilts when moving from the closed mouth position to the maximum open position, tilts to a maximum of 20°, and the Y-axis and Z-axis tilt to a maximum of approximately 7°. The magnetic flux density measured by the magnetic sensor during the movement between the maximum mouth opening and closing is tilted accordingly and increases error. Therefore, it must be corrected by using the measurement data of orientation, as mentioned in Materials and Methods section.

**Figure 16:**
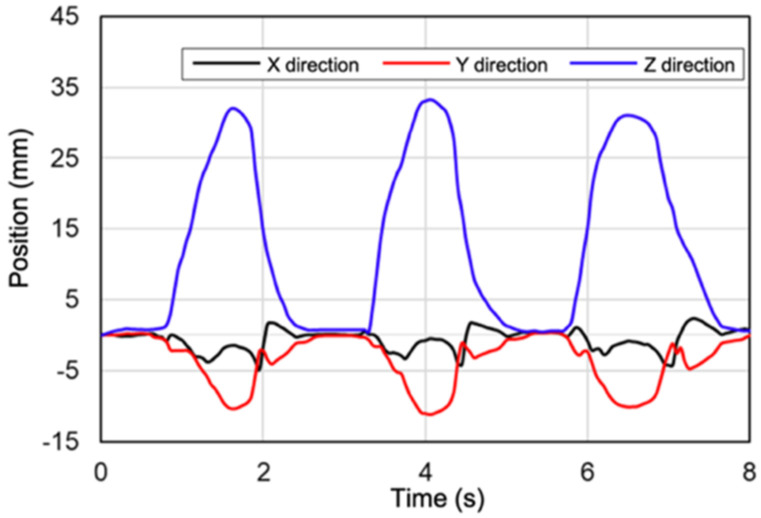
Time development of the estimated position during the patient’s maximum mouth opening.

On account the magnetic flux density and orientation data can be obtained over time by using this method, the relative 3D position and direction of the lower jaw can be obtained (Figure 15b and Figure 16). Therefore, obtaining chronological information in six dimensions of position and orientation becomes possible. This is how it is able to measure phenomena related to the jaw movement that change over time such as frequency and pace of mastication and frequency and degree of bruxism. These novel features will allow going beyond simple assessment of jaw opening and closure to provide information helpful for jaw healthcare in daily life.

**Figure 17:**
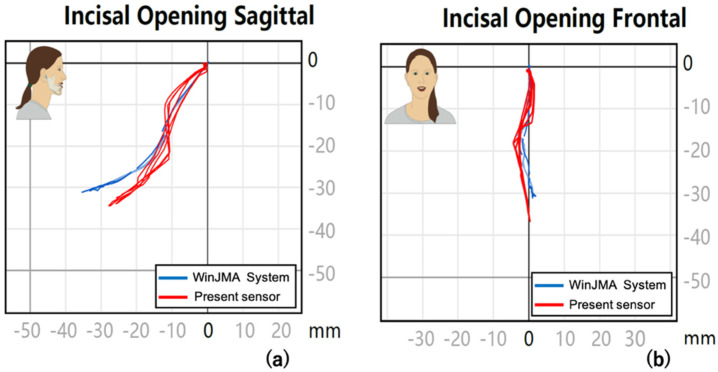
Sagittal and frontal views of the trajectory of jaw movement and trajectory data with simultaneous measurement with the WinJMA System and the present sensor. (**a**) Superimposed trajectories from the sagittal and (**b**) frontal views.

The sagittal and frontal views of the trajectory of jaw movement and trajectory data with simultaneous measurement with the WinJMA System are shown in Figure 17.

The WinJMA System sagittal and frontal views show that the volunteers’ maximum mouth opening occurs at approximately 30 mm from the Z-axis. The value measured by the present sensor is approximately 33 mm (i.e., the error is approximately 3.0 mm). One possible cause of this error is the presence of hysteresis due to residual magnetization. The presumption is that the closing movement does not follow the same trajectory as the opening movement due to hysteresis, thereby resulting in an error of approximately 3.0 mm. A difference of up to ±1.5 mm indeed existed when we compared the position measurement results with and without residual magnetization in the sensor. In addition, a plausible explanation is that the ambient temperature affects the output value of the magnetic sensor, and the error may have occurred because of the difference between the room temperature of 25 °C and the oral temperature of 37 °C where the fingerprints were obtained. However, this error can be improved via a temperature correction to the output value of the magnetic sensor. In the sagittal view, the error between the WinJMA System and the present sensor was minimal, with both following nearly the identical trajectories.

**Figure 18:**
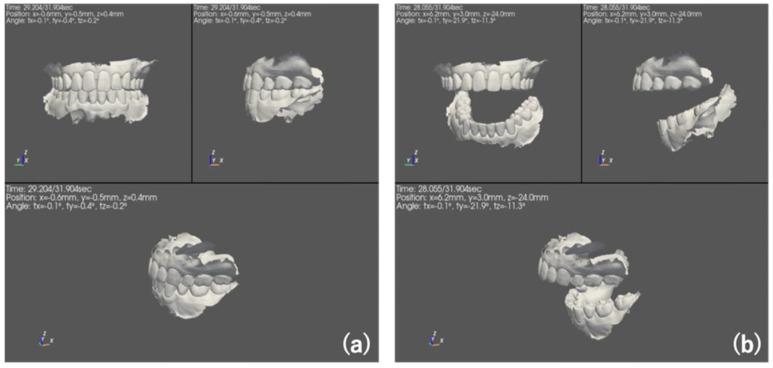
3D reconstruction image at (**a**) mouth closing and (**b**) opening.

Furthermore, we combined the STL data of the volunteers’ dentition obtained with an intraoral scanner and the data measured by this sensor to build a 3D movie by applying this sensor’s mandibular movement tracking with 6 degrees of freedom (i.e., position and tilt) (Figure 18, movie available in Supplementary Materials (Video S1: Three-dimensional images of mandibular movement)). Three-dimensional representations of jaw movement can be effectively used for feedback to patients and for rehabilitation for mandibular position. Moreover, it can be effectively used for various types of analysis and evaluation from a free position, even after it is used for the initial tracking. The simple configuration that allows the sensor and accessories to be placed in the oral cavity also made possible assessing mandibular movement from all directions possible.

## 4. Conclusions

This study demonstrated a tracking method using a small, low-power-consumption composite sensor mounted on a mouthpiece to measure mandibular movement in three dimensions without posing physiological or postural restrictions on the wearer. The sensor can measure with an error within 1.5 mm in the sagittal view and 2.0 mm in the frontal view, up to a maximum mouth opening of approximately 30 mm. The signal-to-noise ratio of the trajectory of the magnetic sensor decreases beyond the maximum mouth opening of 30 mm, thereby increasing error, creating a noisy trajectory, and making measurements difficult to obtain. Thus, the measurement should be taken at the molars, which helps in measuring a mouth opening of approximately 10 mm more and theoretically allows measurements up to approximately 40 mm.

To evaluate the effectiveness of this tracking device, we performed simultaneous measurements using the mouthpiece equipped with this sensor and with the WinJMA system, an existing device for measuring mandibular movement, to perform the opening and closing movements to approximately 30 mm mouth opening. This experiment revealed that the difference between the two devices was small in the sagittal and the frontal views, and they followed nearly the same trajectory. Simultaneous measurement with the WinJMA system and the fingerprint tracking system allowed the trajectory data of mandibular movement with smooth trajectories and small errors to be obtained, which highlights its potential as a novel method of tracking mandibular movement.

The adult male individuals in this study had a mouth opening of approximately 30 mm. However, the average maximum mouth opening of an adult is approximately 50–60 mm, albeit with some individual differences [24]. Such situation is highly likely because of errors and greater scattering the measurement range and improving accuracy by using a magnet with high surface magnetic flux density or a magnetic sensor with higher resolution are necessary to make measuring a mouth opening of ≥40 mm possible. In addition, another challenge of this method is position estimation error because of hysteresis resulting from residual magnetization, which may explain the errors of ≥2.0 mm. An error ≤ 1.0 mm is expected to be achieved by reducing magnetic hysteresis, improving sensor resolution, introducing temperature compensation and strong magnetic flux density by increasing the volume of magnets, and also installing magnets and sensors not only on the left but also on the right side for the differential measurement, which is tolerant to disturbance.

Moreover, the combined and simultaneous use of an orientation sensor and position estimation by a magnetic sensor enabled measuring mandibular movement with six degrees of freedom, which allowed the building of a 3D movement model to evaluate mandibular movement from multiple perspectives. Furthermore, representing arbitrary movements, including the position and tilt of the mandible, is possible; therefore, obtaining the motion trajectory of a single point of the lower jaw is possible, and determining how each part of the mandible is moving and has an effect is possible. For example, indirectly estimating the translational and rotational movements of the mandibular condyle, which are normally difficult to evaluate noninvasively, can aid the study of abnormal jaw movements in more detail.

Existing mandibular movement tracking devices are all large and wired, and therefore may not be tracking natural jaw movement. To overcome the limitations of existing devices, the tracking system proposed in this study used compact magnetic and orientation sensors so that the natural jaw movement could be reproduced. The control board in the configuration proposed in this study was placed extraorally, although selecting a small microcomputer would make possible for the entire system to be self-contained in a small device that fits in the oral cavity to measure mandibular movement. Additionally, its wireless data-sending feature allows tracking to be conducted in a patient’s home to track movements, such as the trajectory of the head in daily living activities and the mandibular position during sleep, that cannot be tracked during visits to medical institutions [25]. Thus, it can be expected to cross the realms of clinical dentistry to provide benefits for physicians and patients in general health management. Although the coatings used in this study are sufficient for short-term experiments, improving the structure to have better biocompatibility (including corrosion resistance) for long-term continuous measurement of patients’ jaw movements is necessary. Various studies have been conducted on the structure of magnets and sensors to be placed safely in the oral cavity [26,27]. Based on these studies, the device will be developed to collect the patient’s long-term daily jaw movement data with safety. In the future, we plan to proceed with the development of a jaw movement measurement method that can be used more easily and widely for obtaining daily biological data in various situations.

## Data Availability

Not applicable.

## Supplementary Materials

The following supporting information can be downloaded at: https://drive.google.com/file/d/1DcAm_tv9drNER1iwB9fGewpBuyg-3iyE/view?usp=sharing (accessed on 16 October 2022). Video S1: Three-dimensional images of mandibular movement.
